# Tetra­kis[2-(benzyl­amino­carbonyl)phenox­ymeth­yl]methane

**DOI:** 10.1107/S1600536808028286

**Published:** 2008-09-13

**Authors:** Xue-Qin Song

**Affiliations:** a School of Chemical and Biological Engineering, Lanzhou Jiaotong University, Lanzhou 730000, People’s Republic of China

## Abstract

The title compound, C_61_H_56_N_4_O_8_, is arranged with all four salicylamide arms disposed in a circular fashion. Each arm has a similar conformation and they are extended so that their terminal groups can fold back. The four carbonyl O atoms are located on the outside of the mol­ecule. The structure is stabilized by intra- and inter­molecular N—H⋯O hydrogen-bonding inter­actions.

## Related literature

For details of the advantages of salicylamide ligands, see: Zhang *et al.* (2002 ▶); Tang *et al.* (2005 ▶). Salicylamide-derived ligands are excellent complexing agents for lanthanide ions because of their attractive sensitization of lanthanide luminescence as well as the construction of novel metal–organic frameworks, see: Song, Dou *et al.* (2007 ▶); Song, Liu *et al.* (2007 ▶); Song *et al.* (2008 ▶). For bond-length data, see: Allen *et al.* (1987 ▶). For related literature, see: Farber & Conley (1974 ▶); Jiri *et al.* (1994 ▶).
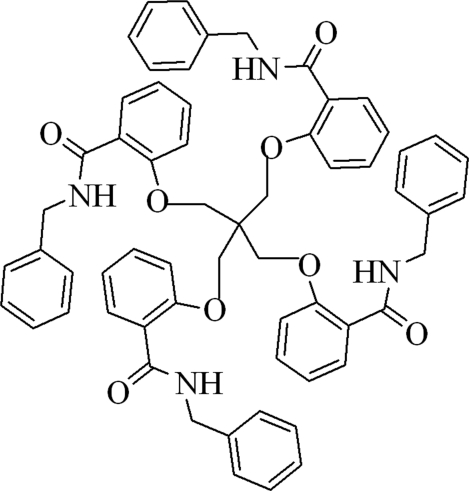


## Experimental

### 

#### Crystal data

C_61_H_56_N_4_O_8_
*M* *_r_* = 973.10
Triclinic, 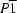

*a* = 13.0505 (4) Å
*b* = 13.6943 (4) Å
*c* = 14.3577 (4) Å
α = 92.519 (1)°
β = 102.677 (1)°
γ = 92.737 (1)°
*V* = 2496.66 (13) Å^3^
*Z* = 2
Mo *K*α radiation
μ = 0.09 mm^−1^
*T* = 153 (2) K
0.39 × 0.36 × 0.10 mm

#### Data collection

Rigaku R-AXIS RAPID diffractometer
Absorption correction: none
20607 measured reflections
9277 independent reflections
7923 reflections with *I* > 2σ(*I*)
*R*_int_ = 0.026

#### Refinement

*R*[*F* ^2^ > 2σ(*F* ^2^)] = 0.041
*wR*(*F* ^2^) = 0.119
*S* = 0.98
9277 reflections
659 parameters
H-atom parameters constrained
Δρ_max_ = 0.33 e Å^−3^
Δρ_min_ = −0.28 e Å^−3^

### 

Data collection: *RAPID-AUTO* (Rigaku, 2004 ▶); cell refinement: *RAPID-AUTO*; data reduction: *RAPID-AUTO*; program(s) used to solve structure: *SHELXS97* (Sheldrick, 2008 ▶); program(s) used to refine structure: *SHELXL97* (Sheldrick, 2008 ▶); molecular graphics: *SHELXTL* (Sheldrick, 2008 ▶); software used to prepare material for publication: *SHELXTL*.

## Supplementary Material

Crystal structure: contains datablocks I, global. DOI: 10.1107/S1600536808028286/at2625sup1.cif

Structure factors: contains datablocks I. DOI: 10.1107/S1600536808028286/at2625Isup2.hkl

Additional supplementary materials:  crystallographic information; 3D view; checkCIF report

## Figures and Tables

**Table 1:** Hydrogen-bond geometry (Å, °)

*D*—H⋯*A*	*D*—H	H⋯*A*	*D*⋯*A*	*D*—H⋯*A*
N1—H1⋯O1	0.88	2.12	2.7705 (16)	130
N2—H2⋯O3	0.88	1.96	2.6731 (14)	137
N3—H3⋯O5	0.88	2.27	2.7399 (14)	113
N4—H8⋯O7	0.88	2.16	2.7341 (16)	122
N4—H8⋯O4^i^	0.88	2.54	3.1841 (17)	131

Symmetry code: (i) 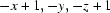.

**Fig. 1.**
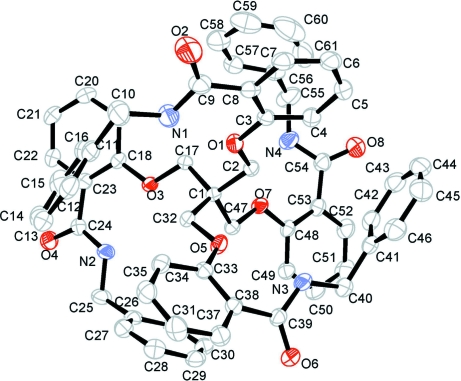
ORTEP plot of the title compound, with the atom-numbering scheme. Displacement ellipsoids are drawn at the 50% probability level. For the sake of clarity, H atoms have been omitted.

## supplementary crystallographic information

### Comment

In the last decade, synthetic studies on salicylamide delived ligands have been carried out because of their attractive sensitization of lanthanide luminescence as well as construction of novel metal–organic frameworks (Zhang *et al.*, 2002; Tang *et al.*, 2005) we are systematically studying the structure and the photophysical properties of complexing salicylamide ligands of capable of sensitizing Eu(III) and Tb(III) emission recently. So far, some intriguing supramolecular complexes based on salicylamide derivatives have been reported (Song, Dou *et al.*, 2007; Song, Liu *et al.*, 2007; Song *et al.*, 2008). However, the tetrapodal ligand featuring salicylamide pendant arms, which is analogues of the ligands studies above, is still unprecedented. In view of this, we report here the synthesis and crystal structure of the title compound (I) which is a part of our studies.

The molecular structure of the title compound (I) is shown in Fig. 1. All bond lengths and angles in (I) are generally within normal ranges (Allen *et al.*, 1987). Four benzylsalicylamide arms are linked together by the quarternary carbon atom at the tetrahedry position and are severely twisted with a *ca* 65° dihedral angle between phenyl rings. The amide hydrogen atoms (H1, H2, H3 and H4) are hydrogen bonded to the ether oxygen atoms (O1, O3, O5 and O7) to provide four hydrogen bonded six-membered rings. It is no doubt to note that the robust intramolecular hydrogen bond have a template effect and participate in the stabilization of the complete structure (Table 1). Interestingly, the title compound was arranged with all four salicylamide arms in a circular fashion and each salicylamide arm had a similar conformation and the arm is so long that their terminal groups can "*fold back*". Four oxygen atoms of the carbonyl groups of the compound, namely, O2, O4, O6 and, O8, are located in the outer part of the whole molecule.

### Experimental

The title compound was synthesized by the following method. To a solution of pentaerythritol benzenesulfonate (2.32 g, 3.3 mmol) (Farber *et al.*,1974) in dry DMF was added K_2_CO_3_ (0.69 g, 5 mmol) and the mixture stirred and heated for 10 min, *N*-benzylsalicylamide (3.40 g, 15 mmol) (Jiri *et al.*,1994) in 50 ml DMF was added dropwise in 30 min and the resulting solution stirred and heated to reflux for 12 h. After cooling down, inorganic salts was separated by filtration and the solvent was removed from the filtrate under reduced pressure. The crude product was purified by chromatography on silica, gradient elution from petroleum to 10:1 petroleum–ethyl acetate to give a white powder. Colourless single crystals were grown from methanol and ethyl acetate mixed solution (v:v =1:5) with slow evaporation at room temperature. The crystals were isolated, washed three times with ethyl acetate and dried in vacuum desiccator using anhydrous CaCl_2_ (yield 87.4%). Analysis found (%): C 75.59, H 5.59, N 5.18; calculated for C_61_H_56_N_4_O_8_: C 75.29, H 5.61, N 5.20.

### Refinement

All H atoms were positioned geometrically and allowed to ride on their parent atoms, with C—H = 0.95 and 0.99 Å and N—H =distance of 0.88 Å, with *U*_iso_(H) = 1.2*U*_eq_(C, N).

### Crystal data

**Table:**

C_61_H_56_N_4_O_8_	*Z* = 2
*M**_r_* = 973.10	*F*(000) = 1028
Triclinic, *P*1	*D*_x_ = 1.294 Mg m^−^^3^
Hall symbol: -P 1	Mo *K*α radiation, λ = 0.71073 Å
*a* = 13.0505 (4) Å	Cell parameters from 19896 reflections
*b* = 13.6943 (4) Å	θ = 3.0–27.5°
*c* = 14.3577 (4) Å	µ = 0.09 mm^−^^1^
α = 92.519 (1)°	*T* = 153 K
β = 102.677 (1)°	Block, colourless
γ = 92.737 (1)°	0.39 × 0.36 × 0.10 mm
*V* = 2496.66 (13) Å^3^	

### Data collection

**Table:**

Rigaku R-AXIS RAPID diffractometer	7923 reflections with *I* > 2σ(*I*)
Radiation source: Rotating Anode	*R*_int_ = 0.026
graphite	θ_max_ = 25.5°, θ_min_ = 3.0°
ω scans	*h* = −15→15
20607 measured reflections	*k* = −16→16
9277 independent reflections	*l* = −14→17

### Refinement

**Table:**

Refinement on *F*^2^	Secondary atom site location: difference Fourier map
Least-squares matrix: full	Hydrogen site location: inferred from neighbouring sites
*R*[*F*^2^ > 2σ(*F*^2^)] = 0.041	H-atom parameters constrained
*wR*(*F*^2^) = 0.119	*w* = 1/[σ^2^(*F*_o_^2^) + (0.0761*P*)^2^ + 0.458*P*] where *P* = (*F*_o_^2^ + 2*F*_c_^2^)/3
*S* = 0.98	(Δ/σ)_max_ = 0.001
9277 reflections	Δρ_max_ = 0.33 e Å^−^^3^
659 parameters	Δρ_min_ = −0.28 e Å^−^^3^
0 restraints	Extinction correction: SHELXL97 (Sheldrick, 2008), Fc^*^=kFc[1+0.001xFc^2^λ^3^/sin(2θ)]^-1/4^
Primary atom site location: structure-invariant direct methods	Extinction coefficient: 0.0058 (9)

### Special details

**Table:**

Geometry. All e.s.d.'s (except the e.s.d. in the dihedral angle between two l.s. planes) are estimated using the full covariance matrix. The cell e.s.d.'s are taken into account individually in the estimation of e.s.d.'s in distances, angles and torsion angles; correlations between e.s.d.'s in cell parameters are only used when they are defined by crystal symmetry. An approximate (isotropic) treatment of cell e.s.d.'s is used for estimating e.s.d.'s involving l.s. planes.
Refinement. Refinement of *F*^2^ against ALL reflections. The weighted *R*-factor *wR* and goodness of fit *S* are based on *F*^2^, conventional *R*-factors *R* are based on *F*, with *F* set to zero for negative *F*^2^. The threshold expression of *F*^2^ > σ(*F*^2^) is used only for calculating *R*-factors(gt) *etc*. and is not relevant to the choice of reflections for refinement. *R*-factors based on *F*^2^ are statistically about twice as large as those based on *F*, and *R*- factors based on ALL data will be even larger.

### Fractional atomic coordinates and isotropic or equivalent isotropic displacement parameters (Å^2^)

**Table:**

	*x*	*y*	*z*	*U*_iso_*/*U*_eq_	
O1	0.71522 (7)	0.40146 (6)	0.74396 (7)	0.0307 (2)	
O2	0.54601 (10)	0.64429 (8)	0.78693 (12)	0.0661 (4)	
O3	0.55519 (6)	0.13763 (6)	0.61990 (6)	0.0280 (2)	
O4	0.34649 (7)	−0.10874 (7)	0.59740 (7)	0.0393 (2)	
O5	0.74512 (6)	0.23924 (7)	0.88530 (6)	0.0311 (2)	
O6	0.96952 (7)	0.15816 (7)	1.09691 (7)	0.0380 (2)	
O7	0.83899 (6)	0.15492 (6)	0.64490 (6)	0.0286 (2)	
O8	1.04764 (10)	0.26366 (12)	0.50380 (11)	0.0738 (4)	
N1	0.54553 (10)	0.48316 (9)	0.79809 (11)	0.0468 (3)	
H1	0.5791	0.4291	0.7962	0.056*	
N2	0.49599 (9)	−0.03234 (8)	0.68268 (8)	0.0327 (3)	
H2	0.5412	0.0186	0.6886	0.039*	
N3	0.95367 (8)	0.25842 (9)	0.97542 (8)	0.0353 (3)	
H3	0.9135	0.2994	0.9409	0.042*	
N4	0.87460 (10)	0.22638 (11)	0.47927 (9)	0.0463 (3)	
H8	0.8248	0.1920	0.4980	0.056*	
C1	0.70914 (9)	0.22638 (9)	0.71619 (8)	0.0239 (3)	
C2	0.77547 (9)	0.32277 (9)	0.72051 (9)	0.0259 (3)	
H2A	0.8416	0.3211	0.7697	0.031*	
H2B	0.7936	0.3323	0.6580	0.031*	
C3	0.76371 (10)	0.49411 (9)	0.75546 (9)	0.0286 (3)	
C4	0.86715 (11)	0.51142 (10)	0.74710 (11)	0.0375 (3)	
H4	0.9068	0.4583	0.7335	0.045*	
C5	0.91243 (12)	0.60600 (11)	0.75858 (12)	0.0443 (4)	
H5	0.9829	0.6173	0.7523	0.053*	
C6	0.85659 (12)	0.68387 (11)	0.77900 (12)	0.0438 (4)	
H6	0.8878	0.7487	0.7868	0.053*	
C7	0.75427 (12)	0.66593 (10)	0.78797 (10)	0.0380 (3)	
H7	0.7158	0.7195	0.8027	0.046*	
C8	0.70542 (10)	0.57233 (9)	0.77623 (9)	0.0309 (3)	
C9	0.59294 (12)	0.56872 (10)	0.78709 (11)	0.0383 (3)	
C10	0.43784 (13)	0.47788 (12)	0.81327 (16)	0.0571 (5)	
H10A	0.3903	0.5062	0.7590	0.068*	
H10B	0.4357	0.5172	0.8723	0.068*	
C11	0.39984 (11)	0.37408 (11)	0.82190 (12)	0.0413 (3)	
C12	0.37785 (11)	0.34375 (12)	0.90675 (12)	0.0438 (4)	
H12	0.3878	0.3890	0.9605	0.053*	
C13	0.34145 (11)	0.24789 (13)	0.91390 (13)	0.0475 (4)	
H13	0.3266	0.2279	0.9723	0.057*	
C14	0.32691 (12)	0.18217 (13)	0.83652 (14)	0.0526 (4)	
H14	0.3025	0.1165	0.8414	0.063*	
C15	0.34770 (13)	0.21148 (13)	0.75205 (14)	0.0537 (4)	
H15	0.3373	0.1660	0.6984	0.064*	
C16	0.38371 (12)	0.30678 (13)	0.74455 (13)	0.0475 (4)	
H16	0.3975	0.3263	0.6856	0.057*	
C17	0.62289 (9)	0.22442 (9)	0.62503 (9)	0.0256 (3)	
H17A	0.5816	0.2832	0.6254	0.031*	
H17B	0.6548	0.2248	0.5685	0.031*	
C18	0.46197 (9)	0.13203 (9)	0.55231 (9)	0.0260 (3)	
C19	0.43425 (10)	0.20619 (10)	0.49037 (9)	0.0318 (3)	
H19	0.4825	0.2605	0.4909	0.038*	
C20	0.33656 (11)	0.20148 (10)	0.42781 (10)	0.0356 (3)	
H20	0.3174	0.2535	0.3871	0.043*	
C21	0.26687 (10)	0.12169 (11)	0.42430 (10)	0.0356 (3)	
H21	0.1994	0.1190	0.3823	0.043*	
C22	0.29651 (10)	0.04574 (10)	0.48274 (9)	0.0312 (3)	
H22	0.2495	−0.0103	0.4780	0.037*	
C23	0.39317 (9)	0.04838 (9)	0.54858 (9)	0.0265 (3)	
C24	0.41076 (10)	−0.03741 (9)	0.61097 (9)	0.0280 (3)	
C25	0.51685 (12)	−0.10807 (10)	0.75153 (10)	0.0394 (3)	
H25A	0.5347	−0.1686	0.7198	0.047*	
H25B	0.4532	−0.1233	0.7763	0.047*	
C26	0.60712 (11)	−0.07326 (9)	0.83336 (10)	0.0315 (3)	
C27	0.58850 (11)	−0.02576 (10)	0.91483 (10)	0.0367 (3)	
H27	0.5184	−0.0146	0.9191	0.044*	
C28	0.67124 (12)	0.00558 (11)	0.98991 (10)	0.0385 (3)	
H28	0.6575	0.0386	1.0451	0.046*	
C29	0.77350 (11)	−0.01078 (10)	0.98518 (10)	0.0372 (3)	
H29	0.8299	0.0094	1.0376	0.045*	
C30	0.79369 (11)	−0.05673 (10)	0.90374 (11)	0.0370 (3)	
H30	0.8640	−0.0676	0.8996	0.044*	
C31	0.71047 (11)	−0.08675 (9)	0.82822 (10)	0.0343 (3)	
H31	0.7245	−0.1171	0.7719	0.041*	
C32	0.66037 (9)	0.22090 (9)	0.80331 (9)	0.0255 (3)	
H32A	0.6075	0.2707	0.8018	0.031*	
H32B	0.6254	0.1553	0.8046	0.031*	
C33	0.72272 (9)	0.24358 (9)	0.97413 (9)	0.0262 (3)	
C34	0.62222 (10)	0.25612 (10)	0.98859 (10)	0.0321 (3)	
H34	0.5649	0.2598	0.9353	0.039*	
C35	0.60552 (11)	0.26319 (11)	1.08030 (10)	0.0367 (3)	
H35	0.5370	0.2733	1.0900	0.044*	
C36	0.68784 (12)	0.25563 (12)	1.15780 (10)	0.0408 (3)	
H36	0.6765	0.2619	1.2209	0.049*	
C37	0.78727 (11)	0.23888 (11)	1.14329 (10)	0.0352 (3)	
H37	0.8430	0.2308	1.1968	0.042*	
C38	0.80706 (10)	0.23364 (9)	1.05186 (9)	0.0275 (3)	
C39	0.91648 (9)	0.21274 (9)	1.04265 (9)	0.0282 (3)	
C40	1.05744 (10)	0.24398 (11)	0.95624 (11)	0.0360 (3)	
H40A	1.0559	0.1808	0.9195	0.043*	
H40B	1.1097	0.2415	1.0174	0.043*	
C41	1.08952 (9)	0.32610 (10)	0.90026 (10)	0.0324 (3)	
C42	1.09177 (10)	0.31052 (12)	0.80485 (10)	0.0379 (3)	
H42	1.0717	0.2475	0.7741	0.046*	
C43	1.12294 (11)	0.38555 (14)	0.75333 (12)	0.0473 (4)	
H43	1.1234	0.3739	0.6877	0.057*	
C44	1.15308 (12)	0.47663 (13)	0.79751 (14)	0.0509 (4)	
H44	1.1753	0.5280	0.7627	0.061*	
C45	1.15102 (13)	0.49342 (12)	0.89261 (14)	0.0520 (4)	
H45	1.1717	0.5565	0.9230	0.062*	
C46	1.11900 (12)	0.41904 (11)	0.94406 (12)	0.0421 (3)	
H46	1.1171	0.4314	1.0093	0.051*	
C47	0.78129 (9)	0.14156 (9)	0.71751 (9)	0.0259 (3)	
H47A	0.8302	0.1409	0.7808	0.031*	
H47B	0.7391	0.0785	0.7051	0.031*	
C48	0.92985 (9)	0.10594 (9)	0.65096 (9)	0.0273 (3)	
C49	0.95748 (10)	0.03237 (10)	0.71371 (10)	0.0325 (3)	
H49	0.9125	0.0132	0.7544	0.039*	
C50	1.05103 (11)	−0.01316 (11)	0.71690 (11)	0.0406 (3)	
H50	1.0695	−0.0640	0.7593	0.049*	
C51	1.11723 (12)	0.01496 (12)	0.65892 (12)	0.0467 (4)	
H51	1.1810	−0.0164	0.6610	0.056*	
C52	1.08965 (11)	0.08932 (12)	0.59773 (11)	0.0427 (4)	
H52	1.1363	0.1097	0.5591	0.051*	
C53	0.99582 (10)	0.13506 (10)	0.59111 (9)	0.0315 (3)	
C54	0.97484 (11)	0.21445 (11)	0.52165 (10)	0.0377 (3)	
C55	0.84401 (15)	0.29339 (17)	0.40364 (12)	0.0637 (5)	
H55A	0.7999	0.2565	0.3469	0.076*	
H55B	0.9080	0.3203	0.3852	0.076*	
C56	0.78409 (12)	0.37703 (13)	0.43166 (10)	0.0441 (4)	
C57	0.67621 (12)	0.36546 (11)	0.41968 (11)	0.0431 (4)	
H57	0.6408	0.3048	0.3937	0.052*	
C58	0.61886 (15)	0.44075 (13)	0.44489 (15)	0.0585 (5)	
H58	0.5447	0.4314	0.4362	0.070*	
C59	0.6685 (2)	0.52872 (14)	0.48233 (16)	0.0722 (6)	
H59	0.6289	0.5799	0.5004	0.087*	
C60	0.7743 (2)	0.54280 (17)	0.49355 (17)	0.0846 (8)	
H60	0.8085	0.6043	0.5183	0.102*	
C61	0.83257 (16)	0.46757 (19)	0.46893 (14)	0.0722 (6)	
H61	0.9066	0.4779	0.4776	0.087*	

### Atomic displacement parameters (Å^2^)

**Table:**

	*U*^11^	*U*^22^	*U*^33^	*U*^12^	*U*^13^	*U*^23^
O1	0.0263 (4)	0.0208 (4)	0.0463 (5)	0.0030 (3)	0.0111 (4)	0.0001 (4)
O2	0.0619 (8)	0.0310 (6)	0.1181 (12)	0.0166 (5)	0.0434 (8)	0.0095 (6)
O3	0.0249 (4)	0.0248 (4)	0.0310 (5)	−0.0007 (3)	−0.0015 (3)	0.0057 (4)
O4	0.0340 (5)	0.0315 (5)	0.0485 (6)	−0.0058 (4)	0.0017 (4)	0.0037 (4)
O5	0.0216 (4)	0.0466 (6)	0.0251 (4)	0.0047 (4)	0.0047 (3)	0.0030 (4)
O6	0.0281 (5)	0.0404 (6)	0.0435 (6)	0.0037 (4)	0.0009 (4)	0.0138 (4)
O7	0.0259 (4)	0.0320 (5)	0.0302 (5)	0.0094 (4)	0.0089 (3)	0.0049 (4)
O8	0.0427 (7)	0.0941 (11)	0.0848 (10)	−0.0071 (7)	0.0081 (6)	0.0503 (8)
N1	0.0374 (7)	0.0287 (6)	0.0814 (10)	0.0114 (5)	0.0253 (6)	0.0092 (6)
N2	0.0340 (6)	0.0263 (5)	0.0348 (6)	−0.0038 (5)	0.0011 (5)	0.0074 (5)
N3	0.0238 (5)	0.0451 (7)	0.0388 (6)	0.0098 (5)	0.0069 (5)	0.0149 (5)
N4	0.0364 (6)	0.0707 (9)	0.0362 (7)	0.0135 (6)	0.0122 (5)	0.0195 (6)
C1	0.0227 (6)	0.0216 (6)	0.0272 (6)	0.0033 (5)	0.0046 (5)	0.0039 (5)
C2	0.0231 (6)	0.0234 (6)	0.0320 (6)	0.0041 (5)	0.0072 (5)	0.0024 (5)
C3	0.0292 (6)	0.0231 (6)	0.0312 (6)	−0.0002 (5)	0.0012 (5)	0.0040 (5)
C4	0.0308 (7)	0.0290 (7)	0.0513 (8)	0.0022 (6)	0.0054 (6)	0.0056 (6)
C5	0.0324 (7)	0.0355 (8)	0.0600 (10)	−0.0055 (6)	−0.0005 (7)	0.0106 (7)
C6	0.0449 (8)	0.0263 (7)	0.0522 (9)	−0.0046 (6)	−0.0062 (7)	0.0070 (6)
C7	0.0453 (8)	0.0253 (7)	0.0392 (8)	0.0041 (6)	−0.0002 (6)	0.0035 (6)
C8	0.0362 (7)	0.0254 (6)	0.0292 (6)	0.0033 (5)	0.0023 (5)	0.0035 (5)
C9	0.0442 (8)	0.0272 (7)	0.0465 (8)	0.0104 (6)	0.0147 (6)	0.0038 (6)
C10	0.0445 (9)	0.0403 (9)	0.0981 (14)	0.0166 (7)	0.0362 (9)	0.0141 (9)
C11	0.0267 (7)	0.0384 (8)	0.0636 (10)	0.0137 (6)	0.0164 (6)	0.0112 (7)
C12	0.0274 (7)	0.0525 (9)	0.0519 (9)	0.0116 (6)	0.0076 (6)	0.0040 (7)
C13	0.0305 (7)	0.0582 (10)	0.0581 (10)	0.0115 (7)	0.0134 (7)	0.0240 (8)
C14	0.0383 (8)	0.0413 (9)	0.0830 (13)	0.0059 (7)	0.0212 (8)	0.0123 (9)
C15	0.0446 (9)	0.0491 (10)	0.0722 (12)	0.0073 (7)	0.0238 (8)	−0.0040 (9)
C16	0.0404 (8)	0.0525 (9)	0.0570 (10)	0.0138 (7)	0.0228 (7)	0.0108 (8)
C17	0.0244 (6)	0.0222 (6)	0.0296 (6)	0.0015 (5)	0.0042 (5)	0.0046 (5)
C18	0.0234 (6)	0.0281 (6)	0.0254 (6)	0.0053 (5)	0.0033 (5)	−0.0015 (5)
C19	0.0337 (7)	0.0295 (7)	0.0300 (7)	0.0027 (5)	0.0018 (5)	0.0029 (5)
C20	0.0386 (7)	0.0348 (7)	0.0306 (7)	0.0101 (6)	−0.0002 (6)	0.0034 (6)
C21	0.0285 (6)	0.0425 (8)	0.0319 (7)	0.0077 (6)	−0.0017 (5)	−0.0025 (6)
C22	0.0253 (6)	0.0346 (7)	0.0324 (7)	0.0017 (5)	0.0051 (5)	−0.0056 (5)
C23	0.0264 (6)	0.0268 (6)	0.0270 (6)	0.0046 (5)	0.0072 (5)	−0.0019 (5)
C24	0.0268 (6)	0.0260 (6)	0.0317 (6)	0.0011 (5)	0.0087 (5)	−0.0021 (5)
C25	0.0453 (8)	0.0296 (7)	0.0391 (8)	−0.0058 (6)	0.0002 (6)	0.0106 (6)
C26	0.0381 (7)	0.0208 (6)	0.0349 (7)	0.0006 (5)	0.0048 (5)	0.0099 (5)
C27	0.0343 (7)	0.0363 (7)	0.0414 (8)	0.0059 (6)	0.0107 (6)	0.0071 (6)
C28	0.0460 (8)	0.0334 (7)	0.0364 (7)	0.0042 (6)	0.0097 (6)	0.0004 (6)
C29	0.0395 (7)	0.0289 (7)	0.0393 (8)	0.0003 (6)	−0.0004 (6)	0.0086 (6)
C30	0.0335 (7)	0.0298 (7)	0.0490 (8)	0.0085 (6)	0.0090 (6)	0.0125 (6)
C31	0.0450 (8)	0.0238 (6)	0.0364 (7)	0.0074 (6)	0.0121 (6)	0.0053 (5)
C32	0.0198 (5)	0.0268 (6)	0.0290 (6)	0.0032 (5)	0.0029 (5)	0.0032 (5)
C33	0.0275 (6)	0.0238 (6)	0.0284 (6)	0.0035 (5)	0.0082 (5)	0.0019 (5)
C34	0.0283 (6)	0.0343 (7)	0.0353 (7)	0.0073 (5)	0.0088 (5)	0.0046 (6)
C35	0.0327 (7)	0.0404 (8)	0.0410 (8)	0.0062 (6)	0.0165 (6)	0.0006 (6)
C36	0.0426 (8)	0.0507 (9)	0.0316 (7)	−0.0025 (7)	0.0158 (6)	−0.0042 (6)
C37	0.0348 (7)	0.0409 (8)	0.0285 (7)	−0.0019 (6)	0.0048 (5)	0.0012 (6)
C38	0.0276 (6)	0.0247 (6)	0.0299 (6)	0.0001 (5)	0.0058 (5)	0.0015 (5)
C39	0.0248 (6)	0.0289 (6)	0.0282 (6)	0.0004 (5)	0.0005 (5)	0.0003 (5)
C40	0.0256 (6)	0.0411 (8)	0.0430 (8)	0.0074 (6)	0.0092 (5)	0.0088 (6)
C41	0.0205 (6)	0.0375 (7)	0.0397 (7)	0.0073 (5)	0.0062 (5)	0.0058 (6)
C42	0.0269 (6)	0.0471 (8)	0.0388 (8)	0.0048 (6)	0.0048 (5)	0.0026 (6)
C43	0.0343 (7)	0.0681 (11)	0.0432 (8)	0.0130 (7)	0.0116 (6)	0.0177 (8)
C44	0.0363 (8)	0.0517 (10)	0.0707 (11)	0.0127 (7)	0.0177 (7)	0.0274 (9)
C45	0.0467 (9)	0.0335 (8)	0.0757 (12)	0.0056 (7)	0.0123 (8)	0.0058 (8)
C46	0.0402 (8)	0.0398 (8)	0.0464 (8)	0.0085 (6)	0.0091 (6)	−0.0004 (7)
C47	0.0242 (6)	0.0253 (6)	0.0293 (6)	0.0050 (5)	0.0071 (5)	0.0033 (5)
C48	0.0226 (6)	0.0261 (6)	0.0317 (6)	0.0037 (5)	0.0035 (5)	−0.0044 (5)
C49	0.0303 (6)	0.0286 (7)	0.0391 (7)	0.0058 (5)	0.0081 (5)	0.0020 (6)
C50	0.0370 (7)	0.0347 (7)	0.0501 (9)	0.0133 (6)	0.0067 (6)	0.0057 (6)
C51	0.0344 (7)	0.0509 (9)	0.0584 (10)	0.0199 (7)	0.0138 (7)	0.0052 (8)
C52	0.0314 (7)	0.0532 (9)	0.0474 (8)	0.0102 (6)	0.0153 (6)	0.0039 (7)
C53	0.0262 (6)	0.0353 (7)	0.0331 (7)	0.0040 (5)	0.0069 (5)	−0.0019 (6)
C54	0.0330 (7)	0.0472 (8)	0.0342 (7)	0.0032 (6)	0.0094 (6)	0.0043 (6)
C55	0.0551 (10)	0.1062 (16)	0.0386 (9)	0.0305 (10)	0.0188 (7)	0.0325 (10)
C56	0.0438 (8)	0.0583 (10)	0.0304 (7)	0.0020 (7)	0.0051 (6)	0.0206 (7)
C57	0.0460 (8)	0.0356 (8)	0.0474 (9)	0.0008 (7)	0.0086 (7)	0.0082 (6)
C58	0.0576 (10)	0.0480 (10)	0.0729 (12)	0.0112 (8)	0.0174 (9)	0.0133 (9)
C59	0.1052 (18)	0.0374 (10)	0.0767 (14)	0.0108 (11)	0.0235 (12)	0.0103 (9)
C60	0.127 (2)	0.0469 (12)	0.0695 (14)	−0.0276 (14)	0.0072 (14)	0.0029 (10)
C61	0.0579 (11)	0.0937 (17)	0.0551 (11)	−0.0284 (12)	−0.0053 (9)	0.0208 (11)

### Geometric parameters (Å, °)

**Table:**

O1—C3	1.3771 (14)	C25—C26	1.5116 (19)
O1—C2	1.4346 (15)	C25—H25A	0.9900
O2—C9	1.2272 (18)	C25—H25B	0.9900
O3—C18	1.3763 (14)	C26—C27	1.387 (2)
O3—C17	1.4359 (14)	C26—C31	1.388 (2)
O4—C24	1.2365 (15)	C27—C28	1.384 (2)
O5—C33	1.3689 (15)	C27—H27	0.9500
O5—C32	1.4312 (14)	C28—C29	1.379 (2)
O6—C39	1.2283 (16)	C28—H28	0.9500
O7—C48	1.3782 (15)	C29—C30	1.385 (2)
O7—C47	1.4267 (14)	C29—H29	0.9500
O8—C54	1.2179 (18)	C30—C31	1.388 (2)
N1—C9	1.3305 (19)	C30—H30	0.9500
N1—C10	1.4677 (19)	C31—H31	0.9500
N1—H1	0.8800	C32—H32A	0.9900
N2—C24	1.3363 (17)	C32—H32B	0.9900
N2—C25	1.4580 (18)	C33—C34	1.3894 (17)
N2—H2	0.8800	C33—C38	1.4015 (17)
N3—C39	1.3374 (17)	C34—C35	1.3811 (19)
N3—C40	1.4605 (16)	C34—H34	0.9500
N3—H3	0.8800	C35—C36	1.378 (2)
N4—C54	1.3372 (19)	C35—H35	0.9500
N4—C55	1.454 (2)	C36—C37	1.387 (2)
N4—H8	0.8800	C36—H36	0.9500
C1—C17	1.5258 (16)	C37—C38	1.3920 (18)
C1—C32	1.5259 (17)	C37—H37	0.9500
C1—C47	1.5280 (17)	C38—C39	1.5023 (17)
C1—C2	1.5349 (16)	C40—C41	1.506 (2)
C2—H2A	0.9900	C40—H40A	0.9900
C2—H2B	0.9900	C40—H40B	0.9900
C3—C4	1.3915 (19)	C41—C42	1.384 (2)
C3—C8	1.4016 (19)	C41—C46	1.393 (2)
C4—C5	1.3853 (19)	C42—C43	1.388 (2)
C4—H4	0.9500	C42—H42	0.9500
C5—C6	1.376 (2)	C43—C44	1.374 (3)
C5—H5	0.9500	C43—H43	0.9500
C6—C7	1.381 (2)	C44—C45	1.381 (3)
C6—H6	0.9500	C44—H44	0.9500
C7—C8	1.3906 (18)	C45—C46	1.385 (2)
C7—H7	0.9500	C45—H45	0.9500
C8—C9	1.509 (2)	C46—H46	0.9500
C10—C11	1.502 (2)	C47—H47A	0.9900
C10—H10A	0.9900	C47—H47B	0.9900
C10—H10B	0.9900	C48—C49	1.3888 (19)
C11—C16	1.384 (2)	C48—C53	1.4003 (18)
C11—C12	1.388 (2)	C49—C50	1.3898 (19)
C12—C13	1.390 (2)	C49—H49	0.9500
C12—H12	0.9500	C50—C51	1.378 (2)
C13—C14	1.372 (3)	C50—H50	0.9500
C13—H13	0.9500	C51—C52	1.383 (2)
C14—C15	1.372 (3)	C51—H51	0.9500
C14—H14	0.9500	C52—C53	1.389 (2)
C15—C16	1.383 (2)	C52—H52	0.9500
C15—H15	0.9500	C53—C54	1.502 (2)
C16—H16	0.9500	C55—C56	1.506 (3)
C17—H17A	0.9900	C55—H55A	0.9900
C17—H17B	0.9900	C55—H55B	0.9900
C18—C19	1.3880 (19)	C56—C57	1.381 (2)
C18—C23	1.4129 (17)	C56—C61	1.394 (3)
C19—C20	1.3859 (18)	C57—C58	1.384 (2)
C19—H19	0.9500	C57—H57	0.9500
C20—C21	1.380 (2)	C58—C59	1.369 (3)
C20—H20	0.9500	C58—H58	0.9500
C21—C22	1.381 (2)	C59—C60	1.359 (4)
C21—H21	0.9500	C59—H59	0.9500
C22—C23	1.3984 (17)	C60—C61	1.387 (4)
C22—H22	0.9500	C60—H60	0.9500
C23—C24	1.5045 (18)	C61—H61	0.9500
			
C3—O1—C2	116.95 (9)	C29—C28—H28	119.8
C18—O3—C17	117.31 (9)	C27—C28—H28	119.8
C33—O5—C32	118.74 (9)	C28—C29—C30	119.75 (13)
C48—O7—C47	117.86 (10)	C28—C29—H29	120.1
C9—N1—C10	120.73 (13)	C30—C29—H29	120.1
C9—N1—H1	119.6	C29—C30—C31	119.47 (13)
C10—N1—H1	119.6	C29—C30—H30	120.3
C24—N2—C25	122.40 (11)	C31—C30—H30	120.3
C24—N2—H2	118.8	C26—C31—C30	121.25 (13)
C25—N2—H2	118.8	C26—C31—H31	119.4
C39—N3—C40	123.53 (11)	C30—C31—H31	119.4
C39—N3—H3	118.2	O5—C32—C1	106.28 (9)
C40—N3—H3	118.2	O5—C32—H32A	110.5
C54—N4—C55	123.08 (14)	C1—C32—H32A	110.5
C54—N4—H8	118.5	O5—C32—H32B	110.5
C55—N4—H8	118.5	C1—C32—H32B	110.5
C17—C1—C32	110.10 (9)	H32A—C32—H32B	108.7
C17—C1—C47	112.88 (10)	O5—C33—C34	122.96 (11)
C32—C1—C47	107.79 (10)	O5—C33—C38	116.52 (11)
C17—C1—C2	106.86 (10)	C34—C33—C38	120.52 (12)
C32—C1—C2	110.77 (10)	C35—C34—C33	120.08 (12)
C47—C1—C2	108.47 (9)	C35—C34—H34	120.0
O1—C2—C1	108.32 (9)	C33—C34—H34	120.0
O1—C2—H2A	110.0	C36—C35—C34	120.25 (13)
C1—C2—H2A	110.0	C36—C35—H35	119.9
O1—C2—H2B	110.0	C34—C35—H35	119.9
C1—C2—H2B	110.0	C35—C36—C37	119.70 (13)
H2A—C2—H2B	108.4	C35—C36—H36	120.1
O1—C3—C4	122.13 (12)	C37—C36—H36	120.1
O1—C3—C8	117.87 (11)	C36—C37—C38	121.35 (13)
C4—C3—C8	120.00 (12)	C36—C37—H37	119.3
C5—C4—C3	120.10 (14)	C38—C37—H37	119.3
C5—C4—H4	120.0	C37—C38—C33	118.00 (12)
C3—C4—H4	120.0	C37—C38—C39	117.74 (11)
C6—C5—C4	120.85 (14)	C33—C38—C39	124.19 (11)
C6—C5—H5	119.6	O6—C39—N3	122.24 (12)
C4—C5—H5	119.6	O6—C39—C38	120.67 (11)
C5—C6—C7	118.67 (13)	N3—C39—C38	117.05 (11)
C5—C6—H6	120.7	N3—C40—C41	110.43 (11)
C7—C6—H6	120.7	N3—C40—H40A	109.6
C6—C7—C8	122.42 (14)	C41—C40—H40A	109.6
C6—C7—H7	118.8	N3—C40—H40B	109.6
C8—C7—H7	118.8	C41—C40—H40B	109.6
C7—C8—C3	117.97 (12)	H40A—C40—H40B	108.1
C7—C8—C9	114.13 (12)	C42—C41—C46	118.69 (14)
C3—C8—C9	127.90 (12)	C42—C41—C40	120.80 (13)
O2—C9—N1	120.31 (14)	C46—C41—C40	120.50 (13)
O2—C9—C8	120.21 (13)	C41—C42—C43	121.04 (15)
N1—C9—C8	119.47 (12)	C41—C42—H42	119.5
N1—C10—C11	111.43 (13)	C43—C42—H42	119.5
N1—C10—H10A	109.3	C44—C43—C42	119.81 (16)
C11—C10—H10A	109.3	C44—C43—H43	120.1
N1—C10—H10B	109.3	C42—C43—H43	120.1
C11—C10—H10B	109.3	C43—C44—C45	119.85 (15)
H10A—C10—H10B	108.0	C43—C44—H44	120.1
C16—C11—C12	118.32 (15)	C45—C44—H44	120.1
C16—C11—C10	120.68 (16)	C44—C45—C46	120.55 (16)
C12—C11—C10	120.99 (16)	C44—C45—H45	119.7
C11—C12—C13	120.70 (16)	C46—C45—H45	119.7
C11—C12—H12	119.7	C45—C46—C41	120.06 (15)
C13—C12—H12	119.7	C45—C46—H46	120.0
C14—C13—C12	119.98 (16)	C41—C46—H46	120.0
C14—C13—H13	120.0	O7—C47—C1	107.87 (10)
C12—C13—H13	120.0	O7—C47—H47A	110.1
C15—C14—C13	119.85 (16)	C1—C47—H47A	110.1
C15—C14—H14	120.1	O7—C47—H47B	110.1
C13—C14—H14	120.1	C1—C47—H47B	110.1
C14—C15—C16	120.37 (17)	H47A—C47—H47B	108.4
C14—C15—H15	119.8	O7—C48—C49	122.89 (12)
C16—C15—H15	119.8	O7—C48—C53	116.60 (11)
C15—C16—C11	120.78 (16)	C49—C48—C53	120.50 (12)
C15—C16—H16	119.6	C48—C49—C50	119.85 (13)
C11—C16—H16	119.6	C48—C49—H49	120.1
O3—C17—C1	109.11 (9)	C50—C49—H49	120.1
O3—C17—H17A	109.9	C51—C50—C49	120.44 (14)
C1—C17—H17A	109.9	C51—C50—H50	119.8
O3—C17—H17B	109.9	C49—C50—H50	119.8
C1—C17—H17B	109.9	C50—C51—C52	119.20 (14)
H17A—C17—H17B	108.3	C50—C51—H51	120.4
O3—C18—C19	121.87 (11)	C52—C51—H51	120.4
O3—C18—C23	117.83 (11)	C51—C52—C53	121.98 (14)
C19—C18—C23	120.29 (11)	C51—C52—H52	119.0
C20—C19—C18	120.37 (12)	C53—C52—H52	119.0
C20—C19—H19	119.8	C52—C53—C48	118.00 (13)
C18—C19—H19	119.8	C52—C53—C54	116.83 (12)
C21—C20—C19	120.46 (13)	C48—C53—C54	125.15 (12)
C21—C20—H20	119.8	O8—C54—N4	122.24 (15)
C19—C20—H20	119.8	O8—C54—C53	120.27 (13)
C20—C21—C22	119.11 (12)	N4—C54—C53	117.46 (12)
C20—C21—H21	120.4	N4—C55—C56	113.42 (13)
C22—C21—H21	120.4	N4—C55—H55A	108.9
C21—C22—C23	122.39 (12)	C56—C55—H55A	108.9
C21—C22—H22	118.8	N4—C55—H55B	108.9
C23—C22—H22	118.8	C56—C55—H55B	108.9
C22—C23—C18	117.27 (12)	H55A—C55—H55B	107.7
C22—C23—C24	115.81 (11)	C57—C56—C61	117.51 (18)
C18—C23—C24	126.87 (11)	C57—C56—C55	119.61 (16)
O4—C24—N2	121.56 (12)	C61—C56—C55	122.88 (17)
O4—C24—C23	120.61 (11)	C56—C57—C58	121.06 (16)
N2—C24—C23	117.79 (10)	C56—C57—H57	119.5
N2—C25—C26	109.82 (10)	C58—C57—H57	119.5
N2—C25—H25A	109.7	C59—C58—C57	120.33 (19)
C26—C25—H25A	109.7	C59—C58—H58	119.8
N2—C25—H25B	109.7	C57—C58—H58	119.8
C26—C25—H25B	109.7	C60—C59—C58	120.0 (2)
H25A—C25—H25B	108.2	C60—C59—H59	120.0
C27—C26—C31	118.45 (12)	C58—C59—H59	120.0
C27—C26—C25	120.67 (13)	C59—C60—C61	120.13 (19)
C31—C26—C25	120.88 (13)	C59—C60—H60	119.9
C28—C27—C26	120.58 (13)	C61—C60—H60	119.9
C28—C27—H27	119.7	C60—C61—C56	121.01 (19)
C26—C27—H27	119.7	C60—C61—H61	119.5
C29—C28—C27	120.45 (14)	C56—C61—H61	119.5
			
C3—O1—C2—C1	177.12 (10)	C33—O5—C32—C1	176.76 (10)
C17—C1—C2—O1	72.69 (12)	C17—C1—C32—O5	−170.75 (9)
C32—C1—C2—O1	−47.24 (13)	C47—C1—C32—O5	65.73 (12)
C47—C1—C2—O1	−165.34 (10)	C2—C1—C32—O5	−52.78 (12)
C2—O1—C3—C4	−1.43 (18)	C32—O5—C33—C34	−16.97 (17)
C2—O1—C3—C8	178.57 (11)	C32—O5—C33—C38	162.40 (11)
O1—C3—C4—C5	179.53 (13)	O5—C33—C34—C35	−177.82 (12)
C8—C3—C4—C5	−0.5 (2)	C38—C33—C34—C35	2.8 (2)
C3—C4—C5—C6	0.5 (2)	C33—C34—C35—C36	−1.5 (2)
C4—C5—C6—C7	0.1 (2)	C34—C35—C36—C37	−1.3 (2)
C5—C6—C7—C8	−0.6 (2)	C35—C36—C37—C38	2.8 (2)
C6—C7—C8—C3	0.6 (2)	C36—C37—C38—C33	−1.4 (2)
C6—C7—C8—C9	−178.86 (14)	C36—C37—C38—C39	−178.50 (13)
O1—C3—C8—C7	179.94 (11)	O5—C33—C38—C37	179.24 (11)
C4—C3—C8—C7	−0.05 (19)	C34—C33—C38—C37	−1.37 (19)
O1—C3—C8—C9	−0.7 (2)	O5—C33—C38—C39	−3.91 (18)
C4—C3—C8—C9	179.34 (14)	C34—C33—C38—C39	175.48 (12)
C10—N1—C9—O2	−2.0 (3)	C40—N3—C39—O6	3.6 (2)
C10—N1—C9—C8	177.15 (15)	C40—N3—C39—C38	−178.52 (12)
C7—C8—C9—O2	13.6 (2)	C37—C38—C39—O6	34.71 (17)
C3—C8—C9—O2	−165.82 (15)	C33—C38—C39—O6	−142.15 (13)
C7—C8—C9—N1	−165.53 (14)	C37—C38—C39—N3	−143.22 (13)
C3—C8—C9—N1	15.1 (2)	C33—C38—C39—N3	39.92 (18)
C9—N1—C10—C11	178.91 (15)	C39—N3—C40—C41	−162.40 (13)
N1—C10—C11—C16	−66.5 (2)	N3—C40—C41—C42	−108.13 (14)
N1—C10—C11—C12	114.90 (17)	N3—C40—C41—C46	72.80 (16)
C16—C11—C12—C13	0.6 (2)	C46—C41—C42—C43	0.2 (2)
C10—C11—C12—C13	179.19 (13)	C40—C41—C42—C43	−178.93 (12)
C11—C12—C13—C14	0.0 (2)	C41—C42—C43—C44	0.6 (2)
C12—C13—C14—C15	−0.4 (2)	C42—C43—C44—C45	−0.8 (2)
C13—C14—C15—C16	0.3 (3)	C43—C44—C45—C46	0.2 (2)
C14—C15—C16—C11	0.3 (2)	C44—C45—C46—C41	0.6 (2)
C12—C11—C16—C15	−0.7 (2)	C42—C41—C46—C45	−0.8 (2)
C10—C11—C16—C15	−179.33 (15)	C40—C41—C46—C45	178.31 (13)
C18—O3—C17—C1	168.19 (10)	C48—O7—C47—C1	160.79 (10)
C32—C1—C17—O3	−56.82 (13)	C17—C1—C47—O7	66.85 (12)
C47—C1—C17—O3	63.67 (13)	C32—C1—C47—O7	−171.34 (9)
C2—C1—C17—O3	−177.18 (9)	C2—C1—C47—O7	−51.36 (12)
C17—O3—C18—C19	0.83 (17)	C47—O7—C48—C49	11.48 (16)
C17—O3—C18—C23	−178.04 (10)	C47—O7—C48—C53	−167.43 (10)
O3—C18—C19—C20	−175.35 (12)	O7—C48—C49—C50	−179.20 (12)
C23—C18—C19—C20	3.49 (19)	C53—C48—C49—C50	−0.33 (19)
C18—C19—C20—C21	−1.9 (2)	C48—C49—C50—C51	0.7 (2)
C19—C20—C21—C22	−1.2 (2)	C49—C50—C51—C52	0.2 (2)
C20—C21—C22—C23	2.8 (2)	C50—C51—C52—C53	−1.6 (2)
C21—C22—C23—C18	−1.24 (19)	C51—C52—C53—C48	2.0 (2)
C21—C22—C23—C24	176.44 (12)	C51—C52—C53—C54	−179.74 (14)
O3—C18—C23—C22	176.99 (11)	O7—C48—C53—C52	177.95 (12)
C19—C18—C23—C22	−1.90 (18)	C49—C48—C53—C52	−0.99 (19)
O3—C18—C23—C24	−0.40 (18)	O7—C48—C53—C54	−0.17 (18)
C19—C18—C23—C24	−179.28 (12)	C49—C48—C53—C54	−179.11 (12)
C25—N2—C24—O4	−2.4 (2)	C55—N4—C54—O8	4.7 (3)
C25—N2—C24—C23	175.58 (12)	C55—N4—C54—C53	−173.51 (15)
C22—C23—C24—O4	6.19 (18)	C52—C53—C54—O8	−29.9 (2)
C18—C23—C24—O4	−176.39 (12)	C48—C53—C54—O8	148.21 (16)
C22—C23—C24—N2	−171.85 (12)	C52—C53—C54—N4	148.30 (14)
C18—C23—C24—N2	5.57 (19)	C48—C53—C54—N4	−33.6 (2)
C24—N2—C25—C26	−169.71 (12)	C54—N4—C55—C56	−114.61 (18)
N2—C25—C26—C27	91.87 (16)	N4—C55—C56—C57	−87.02 (19)
N2—C25—C26—C31	−87.18 (16)	N4—C55—C56—C61	93.6 (2)
C31—C26—C27—C28	−1.3 (2)	C61—C56—C57—C58	−0.7 (2)
C25—C26—C27—C28	179.64 (13)	C55—C56—C57—C58	179.85 (15)
C26—C27—C28—C29	−0.6 (2)	C56—C57—C58—C59	0.1 (3)
C27—C28—C29—C30	1.6 (2)	C57—C58—C59—C60	1.0 (3)
C28—C29—C30—C31	−0.7 (2)	C58—C59—C60—C61	−1.3 (3)
C27—C26—C31—C30	2.19 (19)	C59—C60—C61—C56	0.6 (3)
C25—C26—C31—C30	−178.74 (12)	C57—C56—C61—C60	0.4 (3)
C29—C30—C31—C26	−1.2 (2)	C55—C56—C61—C60	179.81 (17)

### Hydrogen-bond geometry (Å, °)

**Table:**

*D*—H···*A*	*D*—H	H···*A*	*D*···*A*	*D*—H···*A*
N1—H1···O1	0.88	2.12	2.7705 (16)	130.
N2—H2···O3	0.88	1.96	2.6731 (14)	137.
N3—H3···O5	0.88	2.27	2.7399 (14)	113.
N4—H8···O7	0.88	2.16	2.7341 (16)	122.
N4—H8···O4^i^	0.88	2.54	3.1841 (17)	131.

Symmetry codes: (i) −*x*+1, −*y*, −*z*+1.
